# Basal bodies across eukaryotes series: basal bodies in the freshwater planarian *Schmidtea mediterranea*

**DOI:** 10.1186/s13630-016-0037-1

**Published:** 2016-03-19

**Authors:** Juliette Azimzadeh, Cyril Basquin

**Affiliations:** Institut Jacques Monod, CNRS UMR7592/Université Paris Diderot, 15 rue Hélène Brion, 75205 Paris Cedex 13, France

**Keywords:** Planaria, Multiciliated cell, De novo basal body assembly, Basal foot

## Abstract

The freshwater planarian *Schmidtea mediterranea* has recently emerged as a valuable model system to study basal bodies (BBs) and cilia. Planarians are free-living flatworms that use cilia beating at the surface of their ventral epidermis for gliding along substrates. The ventral epidermis is composed of multiciliated cells (MCCs) that are similar to the MCCs in the respiratory airways, the brain ventricles, and the oviducts in vertebrates. In the planarian epidermis, each cell assembles approximately eighty cilia that beat in a coordinate fashion across the tissue. The BBs that nucleate these cilia all assemble de novo during terminal differentiation of MCCs. The genome of the planarian *S. mediterranea* has been sequenced and efficient methods for targeting gene expression by RNA interference are available. Defects induced by perturbing the expression of BB proteins can be detected simply by analyzing the locomotion of planarians. BBs are present in large numbers and in predictable orientation, which greatly facilitates analyses by immunofluorescence and electron microscopy. The great ease in targeting gene expression and analyzing associated defects allowed to identify a set of proteins required for BB assembly and function in planarian MCCs. Future technological developments, including methods for transgenic expression in planarians and in related species, will achieve turning free-living flatworms into powerful model systems to study MCCs and the associated human pathologies.

## The organism

**Fig. 1:**
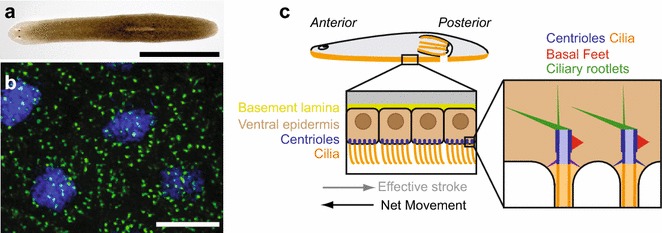
The ventral multiciliated epidermis of planarians. **a** *Schmidtea mediterranea*. *Bar* is 5 mm. **b** Immunofluorescence view of the ventral epidermis of *S.* *mediterranea.* BBs are in *green* (anti-SMED-CEP135) and nuclei are in *blue* (DAPI). *Bar* is 5 µm. **c** Schematic representation of the planarian ventral epidermis

Planarians belong to the phylum Platyhelminthes, or flatworms. Together with mollusks, annelids, and several other groups, flatworms form a major grouping of protostome animals called Lophotrochozoa, which remains little explored with the modern tools of biology. The common name planarian has different significations but often it designs the order Tricladida, which comprises free-living flatworms found in marine, freshwater, or terrestrial environments. Planarians are flat, soft-bodied animals that can be less than 1 mm up to a few cm in length. They are best known for their extraordinary regeneration capacity, being able to form whole animals from even minuscule body fragments [23, 28]. The first systematic studies on planarian regeneration were done by Harriet Randolph and Thomas Hunt Morgan in the late nineteenth century, but experimental work on this topic had been going on for more than a century before that [21, 26]. Another interesting feature of planarians is that they use motile cilia for locomotion. The ventral epidermis is composed of multiciliated cells (MCCs) very similar to those lining the respiratory tract, the ependyma, and the oviducts in vertebrates. In particular, basal bodies (BBs) in vertebrate and planarian MCCs are decorated by similar appendages (see below) [5, 18]. Other aspects of MCC differentiation, such as the involvement of the planar cell polarity pathway in controlling BB docking at the plasma membrane are also conserved between these systems, which suggests a common evolutionary origin of MCCs in bilaterian animals [2, 7]. Planarians deposit a layer of mucus on the substrate and epidermal cilia beat within this layer to propel the animal. Besides the ventral epidermis, the epithelium that lines the feeding organ of planarians, called pharynx, is also multiciliated. In addition, individual MCCs called flame cells are found in the excretory system of planarians, which consists of branched epithelial tubules (called protonephridia) that are present throughout the body. Flame cells at the proximal end of protonephridia allow the ultrafiltration of extracellular fluid as well as fluid circulation driven by ciliary beating [30, 36, 40]. Finally, cilia are found in a subset of sensory neurons and in sperm cells [8, 14, 16]. There are many species of planarians, but most recent studies use a freshwater planarian called *Schmidtea mediterranea* (Fig. 1a). The genome of *S. mediterranea* has been sequenced, and gene inactivation by RNA interference (RNAi) works very efficiently in this species [22, 31, 35].

## Basic basal body structure

**Fig. 2:**
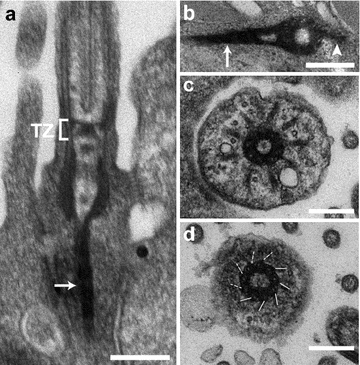
Ultrastructure of BBs and associated structures in *S. mediterranea*. Transmission electron microscope views of BBs in (**a**, **c**, **d**) sensory neurons and (**b**) epidermal MCCs. **a** Longitudinal view of a BB in an epidermal process emanating from a subepidermal sensory neuron. *TZ* transition zone, *arrow* ciliary rootlet. **b** Transverse view of a BB showing the basal foot (*arrowhead*) and the horizontal ciliary rootlet (*arrow*). **c** Transverse view of a BB in a sensory process showing nine**-**fold symmetrical blade-like structures. **d** Transverse section through the same BB as in (**c**) 100 nm away toward the distal end showing the distal appendages (also called transition fibers). *Solid lines* are used to highlight the distal appendages, *dotted lines* are used when the distal appendages are not clearly visible on this section. *Bar* is 0.2 µm in (**a**–**d**)

Basal body (BB) structure has been described in the epidermis of *S. mediterranea* and *Girardia tigrina* [4, 16]. The ventral epidermis of planarians is composed by MCCs that each assembles approximately 80 centrioles (Fig. 1b, c) [4]. In addition, sensory neurons located in the subepidermal parenchyma send cytoplasmic processes that project to the epidermal surface and are terminated by one or two sensory cilia. All epidermal cilia contain a 9 + 2 axoneme, even though sensory cilia display structural specificities such as an increased diameter [16]. Whether sensory cilia are motile or not is unknown. The BBs associated to either MCC or sensory cilia appear very similar. BBs are relatively short, about 250–300 nm long (Fig. 2a) and they are formed by triplet microtubules [4, 16]. Epsilon and delta tubulins, which are involved in the assembly of triplet microtubules in other species, are conserved and essential to BB assembly in planarians [4, 11–13]. Depletion of either gene by RNAi drastically reduces the number of BBs, supporting that epsilon- and delta-tubulin are critical to BB assembly and/or stability [4]. Whether their depletion specifically affects the assembly of microtubule triplets is not known. BBs are attached to a transition zone that contains a thin plate associated to a small cylindrical structure of unknown composition, which is also present in ciliated sensory neurons (Fig. 2a) [16].

## Additional BB structures or accessory structures

Like in vertebrate MCCs, BBs in planarian MCCs have a basal foot (Fig. 2b), an appendage required for controlling centriole rotational polarity and thus the direction of ciliary beat [5, 18]. In addition, BBs in epidermal MCCs are decorated by two ciliary rootlets: a long, vertical rootlet and a short horizontal rootlet (Figs. 1c, 2b). Both rootlets attach to the proximal end of the BB, opposite to the basal foot [5]. In sensory neurons, BBs do not have a basal foot but instead are decorated by blade-like nine-fold symmetrical appendages (Fig. 2c; see also [16]). The function and composition of these appendages are not known but they are possibly related to the subdistal appendages that decorate the mother centriole in vertebrate centrosomes [25]. Sensory BBs are decorated by a single, vertical ciliary rootlet (Fig. 2a). Interestingly, this rootlet shows a slightly different striation pattern than in MCCs [16].

## BB origins

All cell types in *S. mediterranea*—including ciliated cell types—assemble from a population of adult stem cells called neoblasts, which are devoid of centrioles [4, 23, 28]. In this species and probably other planarian species as well, the BBs are always associated to a ciliary axoneme, and never have the function of a centrosome. BBs are assembled de novo during terminal differentiation of ciliated cells from neoblast progenies. The process has not been well documented in planarians, but in other flatworms BBs assemble in the vicinity of clusters of fibrous granules [10]. This is reminiscent of what was observed in other phyla such as Ctenophores, but distinct from vertebrate MCCs in which centrioles form around spherical structures termed deuterosomes [1, 9, 38, 39]. Not all planarian ciliated cell types assemble large number of centrioles, however. Like other flatworms, sexual planarians form spermatocytes with only two BBs, which template the assembly of two motile flagella with a 9 + ′1′ axoneme, in which the central structure is not a microtubule [15]. The two BBs assemble in the vicinity of a multilayered structure called the intercentriolar body during early spermiogenesis [8, 14]. BB assembly in sensory neurons has not been characterized, and it is not even clear whether these cells form single or multiple ciliated processes.

## Identification of BB components

Many components required for BB assembly in planarian MCCs have been identified by RNAi screening [4]. Most human centrosome components have orthologs encoded in the *S. mediterranea* genome, and most of these conserved genes are required for centriole assembly or function. When ciliary assembly is inhibited, planarians only use inchworming locomotion, a slow locomotion mode based on muscle contraction. In addition, the flatworms bloat due to inhibition of ciliary function in flame cells, which leads to defective osmoregulation and edema formation [27, 29, 33, 40]. Proteins essential to BB assembly in planarians include the orthologs of Plk4, CEP152, SAS-6, SAS-5/Ana2, SAS-4, CEP135, CEP120, epsilon-tubulin, delta-tubulin, Ana1, Ana3/Rotatin, and HYLS1. Depleting the orthologs of Ofd1, centrin 2, MKS1, and uncharacterized protein CEP78 also strongly impacts ciliogenesis, apparently by inhibiting BB docking. In addition, depletions of several putative BB components perturb ciliary function by decreasing locomotion speed without inducing inchworming. Among them, an uncharacterized protein called WDR67 was also found to also inhibit ciliogenesis in human RPE-1 cells. Overall, these results show that de novo assembly of BBs in planarian MCCs depends upon the same molecular components than centriole duplication at the mammalian centrosome [4]. Most of these genes are also overexpressed during MCC differentiation in *Xenopus* [19], suggesting a general conservation of the mechanisms underlying BB/centriole assembly. The initial step of BB assembly in MCCs is likely different between planarians and vertebrates, however. In vertebrates, BBs assemble on deuterosomes, which themselves are formed at the centrosome [1]. Deuterosome assembly depends on a protein called Deup1, which is the paralog of CEP63, a protein required for centriole duplication at the centrosome [37, 42]. Both Deup1 and CEP63 bind CEP152, a key regulator of centriole/BB assembly. Depletion of the single CEP63/Deup1 ortholog encoded in the *S. mediterranea* genome has no effect on ciliary-based locomotion however, even though planarian CEP152 depletion strongly inhibits BB assembly [4]. The different requirement for CEP63/Deup1 proteins between planarians and vertebrates might be linked to the fact that BBs in *S. mediterranea* assemble independently of a centrosome, since this organelle is absent in this species.

## Notable BB findings

Planarians have been only very recently used to study BBs, but this has helped identify a set of proteins required for BB assembly and function in MCCs, including previously uncharacterized proteins. All the BB components analyzed are orthologous to components of the human centrosome, many of which were also shown to be required for centriole duplication [3, 24]. This helped establishing that de novo BB assembly in MCCs and centriole duplication rely on similar molecular mechanisms [4]. In mammalian MCCs, the assembly of deuterosomes, on which BBs are formed, is seeded at the centrosome [1]. Planarians do not have centrosomes, and thus BB assembly truly occurs de novo. Nevertheless, most of the key players for centriole duplication are required for de novo BB assembly in planarians. The finding that these animals completely lack centrosomes is helpful for understanding the evolution of this organelle. Firstly, it points to key proteins in the evolution of centrosome function, as centrosome loss is correlated to the loss of genes coding for SPD-2/CEP192 and centrosomin/CDK5RPA2-related proteins. Secondly, the absence of centrosomes in these rather complex organisms suggests that centrosome evolution in animals is linked to its involvement in specific developmental processes like for instance-oriented cell divisions [4]. How the microtubule cytoskeleton organized in freshwater planarians remains little understood. During mitosis, the spindle poles are formed by accumulation of granular material in the vicinity of the plasma membrane [4, 34]. Planarian wild-type spindle poles share similarities with spindle poles of *Drosophila* mutant cells devoid of centrosomes—general aspect in electron micrographs, the absence of astral microtubules, and proximity to the plasma membrane, suggesting that spindle poles could be formed by conserved regulators of spindle assembly [6].

## Strengths and future of BB research in planaria

An interesting feature of planarians is that BB defects induced by gene perturbation can be detected by direct observation of the associated locomotion phenotypes [5, 32]. Measuring locomotion speed is straightforward and it allows identifying even subtle perturbations of ciliary function induced by depletion of BB components. Planarians can be grown easily and inexpensively in the laboratory, and the genome sequence of *S. mediterranea* is available [31]. RNAi works very efficiently in planarians by feeding or injection of long double-strand RNA, and planarians are thus amenable to large-scale RNAi screening [22, 27, 35]. Furthermore, the composition of BBs in planarians is very similar to vertebrates, and studies in planarians are thus relevant for understanding human ciliopathies [4]. This is true for syndromes affecting the function of the respiratory muco-ciliary epithelium such as primary ciliary dyskinesia, but also for kidney disorders such as cystic kidney diseases (CKD). The organization and function of planarian protonephridia and vertebrate nephrons are indeed similar in many respects, and perturbing ciliary functions in planarians induces the formation of cysts in protonephridial tubules that share many features with CKD cysts [40]. Finally, MCCs assemble numerous BBs in predictable orientation with respect to the whole animal, which greatly facilitates analysis of BB ultrastructure by electron microscopy. The main limitation in using planarians for the study of BBs is the fact that reproducible methods for transgenic expression are not yet available. It is however already possible to express fluorescent markers such as GFP in another flatworm, the marine species *Macrostomum lignano* [20]. *M. lignano* locomotion relies on MCCs that cover the entire surface of its epidermis. It is transparent and only about 1.5 mm in length, which is convenient for fluorescence microscopy. The genome sequence is publicly available, and gene silencing by RNAi works very efficiently by simply soaking the animals in dsRNA [17, 41]. *M. lignano* thus constitutes a promising model for studying BBs in MCCs.

## Abbreviations

BB: basal body
CKD: cystic kidney disease
GFP: green fluorescent protein
MCC: multiciliated cell
RNAi: RNA interference
